# A physical model of cell metabolism

**DOI:** 10.1038/s41598-018-26724-7

**Published:** 2018-05-29

**Authors:** Jorge Fernandez-de-Cossio-Diaz, Alexei Vazquez

**Affiliations:** 1 Center of Molecular Immunology, Havana, Cuba; 2 Cancer Research UK Beatson Institute, Glasgow, UK; 3 Institute for Cancer Sciences, University of Glasgow, Glasgow, UK

## Abstract

Cell metabolism is characterized by three fundamental energy demands: to sustain cell maintenance, to trigger aerobic fermentation and to achieve maximum metabolic rate. The transition to aerobic fermentation and the maximum metabolic rate are currently understood based on enzymatic cost constraints. Yet, we are lacking a theory explaining the maintenance energy demand. Here we report a physical model of cell metabolism that explains the origin of these three energy scales. Our key hypothesis is that the maintenance energy demand is rooted on the energy expended by molecular motors to fluidize the cytoplasm and counteract molecular crowding. Using this model and independent parameter estimates we make predictions for the three energy scales that are in quantitative agreement with experimental values. The model also recapitulates the dependencies of cell growth with extracellular osmolarity and temperature. This theory brings together biophysics and cell biology in a tractable model that can be applied to understand key principles of cell metabolism.

## Introduction

Cell metabolism is a network of biochemical reactions transforming metabolites to fulfill biological functions. At the core of this biochemical network there are catabolic pathways that break down molecules to generate energy, which is then used to fuel biosynthetic processes and to do mechanical work. Based on empirical observations there are three major scales of energy demand depending on the cell physiological state.

The basal metabolic state of a cell is characterized by a maintenance energy demand^1,2^. It is currently assumed that the maintenance energy represents the energy cost incurred to maintain ions balance between the cell and the extracellular medium. It is also assumed that the maintenance energy demand is a constant independent of the cell growth rate. In fact, the maintenance energy demand is often estimated from the extrapolation of the growth dependence of the energy demand to the zero growth limit. For mammalian cells it is particularly high, with values around 0.3 mol ATP/L/h^3^.

When cells grow, move or perform other functions the energy requirements increase beyond the basal maintenance demand. Cells utilize glycolysis and oxidative phosphorylation to satisfy these energetic demands. Glycolysis has a low yield of 2 mol ATP/mol glucose^4^, but it is characterized by a high horsepower (energy produced per volume of enzymes)^3,5^. Oxidative phosphorylation has a higher yield of 32 mol ATP/mol glucose^4^, but it is characterized by a lower horsepower^3,5^. The differences in yield and horsepower imply a metabolic switch from pure oxidative phosphorylation at low energy demands to mixed oxidative phosphorylation plus obligatory fermentation (glycolysis + lactate release) at high energy demands^5,6^. For mammalian cells this takes place at an energy demand of about 2 mol ATP/L/h^6^, 10 times the energy demand of cell maintenance.

Finally, there is the energy demand necessary to sustain the maximum growth rate, or maximum metabolic rate in general. The energy demand at maximum growth rate can only be sustained by glycolysis^5^ and therefore we can estimate the maximum energy requirements of cells from their maximum reported rates of fermentation. For mammalian cells that gives us an estimate of about 8 mol ATP/L/h^6^, close to an order of magnitude above the energy threshold for obligatory fermentation.

We currently have a good theoretical understanding of the metabolic switch to aerobic fermentation and the maximum metabolic rate. The key point is that cell metabolism operates within the context of an intracellular milieu crowded with macromolecules and organelles^7^. Molecular crowding imposes a limit on the maximum density of metabolic enzymes and other cellular macromolecules, therefore imposing a limit on maximum metabolic rate^8^. Molecular crowding also implies a metabolic switch from unconstrained enzyme density at low metabolic rates to constrained enzyme density at high metabolic rates, which results in a change of metabolic efficiency with increasing metabolic rate^9^. At low enzyme density cells can increase the density of metabolic enzymes to increase metabolic rate. In this context it is more efficient to use pathways with high yield, explaining why oxidative phosphorylation is preferred to fermentation. However, high metabolic rates require a high enzyme density that is limited by macromolecular packing constraints. In this context efficiency can be quantified as pathway rate per enzyme volume and we speak of molecular crowding cost^8,9^. Furthermore, larger enzymes imply a higher biosynthetic cost and, therefore, another measure of efficiency is the pathway rate per enzyme amino acid^10,11^. Today enzymatic costs are an integral component of cell metabolism models^12–15^.

Yet, no theory has been proposed to explain the origin and magnitude of the maintenance energy demand. Here we address this problem based on the hypothesis that the maintenance energy demand corresponds to the energy expended by molecular motors to fluidize the intracellular milieu. The incorporation of this idea into a basic model of cell metabolism allows us to make quantitative predictions of the three energy scales that are in agreement with measured values. We discuss several implications of this theory to motivate further experimental work for its verification.

## Results

### Entropic pressure of molecular crowding

**Figure 1:**
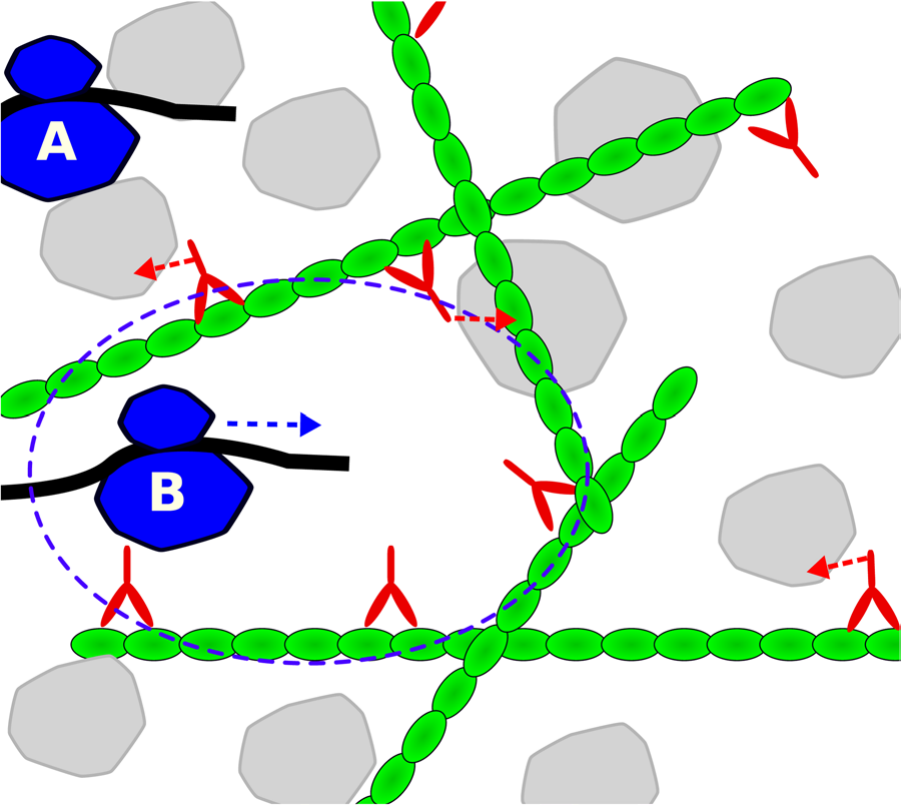
Molecular motors counteracting molecular crowding. Molecular crowders (gray) impede cellular processes such as protein synthesis. (**A**) A ribosome (blue) translating a mRNA strand must displace the crowders before it can move to the next codon. (**B**) Molecular motors (red) increase the propensity of cavities with lower density of molecular crowders where processes like translation can proceed freely.

The machinery of life operates on the background of a gel like substance with properties distinct from ideal solutions^16^. In turn, the properties of this background substance are affected by the molecular crowding of the cellular machinery^17^. Molecular crowding hinders the diffusion of large macromolecules. When a trace particle is confined by the surrounding molecules, impeding its movement, a hole needs to be created to make space available for the trace molecule to diffuse (Fig. 1, molecule A). However, the creation of a hole lowers the entropy of the surrounding system, by reducing the number of microscopic configurations of crowding molecules and free space.

To derive a quantitative expression, we consider a regular lattice of *N* sites where *n* hard spheres can be placed. Volume exclusion is taken into account by allowing a maximum of one sphere per site. The entropic pressure associated with the creation of a hole of size equal to *m* sites can be estimated as (Methods),

1 $$\begin{array}{rcl}{P}_{S} & = & \frac{T{\rm{\Delta }}S}{{V}_{h}}\\  & = & \frac{{k}_{{\rm{B}}}T}{{V}_{h}}[\mathrm{ln}(\begin{array}{c}N\\ n\end{array})-\,\mathrm{ln}(\begin{array}{c}N-m\\ n\end{array})]\\  & \approx  & \frac{{k}_{{\rm{B}}}T}{{V}_{c}}ln\frac{{\rm{\Phi }}}{{\rm{\Phi }}-\varphi }\end{array}$$

where *V*_*c*_ is the typical volume of molecular crowders (the hard spheres), *V*_*h*_ = *mV*_*c*_ the hole volume, *ϕ* = *nV*_*c*_/*V* the excluded volume fraction by the molecular crowders, Φ = *NV*_*c*_/*V* the maximum packing density and *V* the cell volume. The approximation in the last step assumes that $N,n\gg m$ and that $N,N-n\gg 1$, *i.e*., that the space occupied by a single molecular crowder is negligible compared to the total volume of molecular crowders. As expected the entropic pressure diverges as *ϕ* approaches the maximum packing density Φ, indicating that at maximum packing the creation of holes becomes unfeasible. We also note that the entropic pressure is inversely proportional to the crowder volume *V*_*c*_. In deriving the entropic pressure equation (1) we have made some assumptions that may not be valid in general. However, we feel that this logarithmic dependency holds beyond the lattice model considered here. In other words, we can interpret equation (1) as a postulate of this theory. This is an important point to bear in mind, because all the volume fraction dependencies derived below follow directly from this logarithmic dependency. Therefore, deviations from this logarithmic dependency would require us to revisit the calculations reported below.

### Molecular motors pressure

A fundamental question in cell biology is how cells can achieve high metabolic rates in the context of the highly crowded cellular environment. The experimental evidence tells us that high rates of metabolism coincide with a fluidization of the cytoplasm^18^. The fluidization of the cell milieu is most likely determined by the activity of molecular motors that push macromolecules at the expense of ATP hydrolysis^19^. This observation prompted us to the hypothesis that the maintenance energy demand may be rooted on the energy expended by molecular motors. To put this hypothesis into a working model we postulate an ideal gas of molecular motors and determine the motors pressure (Fig. 1, red molecules). As in an ideal gas, we assume the particles (motors) move in a straight line at constant speed, which is a good approximation for the movement of motors along actin filaments. The key difference is that the motors have low speeds compared to ideal gas molecules. However, motors can apply significant forces (kicks) of the order of pN. When motors get in contact with macromolecules they can transmit a large impulse (Fig. 1, arrows). Introducing this modification to the classical kinetic theory of gases we obtain the motors pressure (Methods),

2 $${P}_{M}=\frac{pFd}{12}{n}_{M}$$

where *F* and *d* are the motors force and displacement per kick, *n*_*M*_ is the number of molecular motors per cell volume, and *p* is the motors persistence, a non-dimensional parameter quantifying the motors tendency to maintain their direction of motion upon contact with macromolecules.

The cell could tune the motors concentration in order to counteract the entropic pressure of molecular crowding. The optimal motors concentration should be a balance between the tendency of molecular motors to open holes and facilitate movement (Fig. 1, molecule B) and the energetic cost of motors activity. We postulate that the optimal solution occurs when the motors pressure matches the entropic pressure of molecular crowding *P*_*M*_ = *P*_*S*_. From this postulate and equations (1) and (2) we obtain the volume fraction occupied by molecular motors, *ϕ*_*M*_ = *V*_*M*_*n*_*M*_,

3 $${\varphi }_{M}=\frac{{V}_{M}}{{V}_{c}}\frac{{k}_{{\rm{B}}}T}{{E}_{M}}\,\mathrm{ln}\,\frac{{\rm{\Phi }}}{{\rm{\Phi }}-\varphi }$$

where *V*_*M*_ is the typical volume of molecular motors and *E*_*M*_ = *pFd*/12 is the average motors energy per kick. Within this framework, the volume fraction of molecular motors is proportional to the ratio of the thermal energy *k*_B_*T* to the motors energy per kick *E*_*M*_. It is also evident that the volume fraction of molecular motors is a monotonically increasing function of the macromolecular fraction *ϕ*.

The motors pressure derived above (3) may apply to more general scenarios where molecular motors transmit impulse to macromolecules by other mechanisms. For example, in the process of assisting protein folding, heat shock proteins may transmit impulse to neighbor macromolecules. In general terms, any macromolecule undergoing structural changes driven by ATP or GTP hydrolysis may transmit impulse to their neighbor macromolecules and therefore contribute to the molecular motors pressure. The key factors are the proportionality between the motors pressure and their concentration and what is the typical value of *E*_*M*_.

### Basic model of cell metabolism

Now we are ready to investigate a model of cell metabolism where the maintenance energy demand represents the energy expended by molecular motors to counteract molecular crowding. Since proteins are the major component of cell biomass, we will focus on protein metabolism for the sake of simplicity. Furthermore, since we want to investigate the impact of extracellular medium osmolarity, we will formulate our model in terms of the volume allocation constraint^5,8^. This formulation is more appropriate than the commonly used protein allocation constraint^11^, because it can deal with scenarios where the protein density is variable.

**Figure 2:**
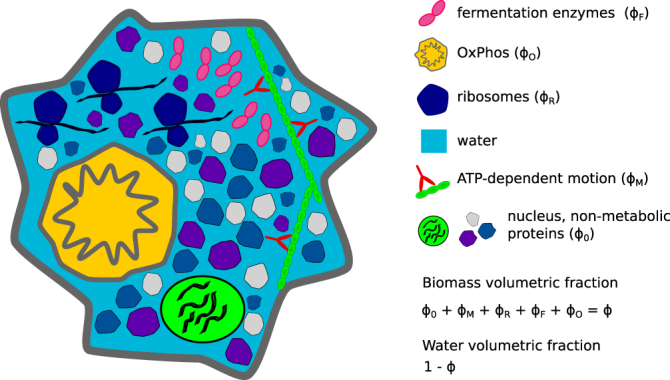
Volumetric partition of cell components. Cell components are classified into 5 groups: (M) proteins driving ATP-dependent motion, (R) ribosomes, (F) fermentative enzymes, (O) oxidative phosphorylation, (0) nucleus and other non-metabolic proteins, containing all components not included in previous groups. The respective volumetric fractions are denoted by *ϕ*_*M*_, *ϕ*_*R*_, *ϕ*_*F*_, *ϕ*_*O*_ and *ϕ*_0_, respectively, while the total volume fraction occupied by biomass in the cell is denoted by *ϕ*. In the case of eukaryotic cells, oxidative phosphorylation occurs in mitochondria (depicted here) and ATP-dependent motion is carried out by molecular motors supported on the cytoskeleton (also shown). In the case of prokaryotic cells, oxidative phosphorylation takes place in the cell membrane (not depicted here).

We divide proteins/organelles into five classes based on their function (Fig. 2): background proteins (*ϕ*_0_), ribosomes (*ϕ*_*R*_), molecular motors (*ϕ*_*M*_), fermentation enzymes (*ϕ*_*F*_) and oxidative phosphorylation machinery (*ϕ*_*O*_). We further assume that cells are in an exponential growth phase, with growth rate *μ*. This simplified model is governed by the equations,

4 $$\varphi =\sum _{i=0,M,R,F,O}{\varphi }_{i}$$

5 $$C=\sum _{i=\mathrm{0,}M,R,F,O}{c}_{i}{\varphi }_{i}$$

6 $${f}_{C}=\frac{{h}_{F}}{{\gamma }_{F}}{\varphi }_{F}+\frac{{h}_{O}}{{\gamma }_{O}}{\varphi }_{O}+\frac{\mu }{{\gamma }_{aa}}C$$

7 $${f}_{M}={m}_{M}{\varphi }_{M}$$

8 $${f}_{E}={h}_{F}{\varphi }_{F}+{h}_{O}{\varphi }_{O}={f}_{M}+{e}_{P}{h}_{R}{\varphi }_{R}$$

9 $${h}_{R}{\varphi }_{R}=({k}_{P}+\mu )C$$

10 $$Y=\frac{\mu C/{\gamma }_{aa}}{{f}_{C}}$$

where *i* = 0, *M*, *R*, *F*, *O* is the compartment index, *ϕ*_*i*_ the volume fraction of compartment *i*, *C* the protein concentration in the cell (mol of amino acids/cell volume), *c*_*i*_ the protein concentration in compartment *i* (mol of amino acids/compartment volume), *k*_*P*_ the protein turnover rate (amino acids/unit of time), *h*_*R*_ the ribosome horsepower (moles of amino acid added to new proteins/volume of ribosome/unit of time), *h*_*F*_ and *h*_*O*_ the fermentation and oxidative phosphorylation horsepowers (moles of ATP/volume of enzyme/unit of time), *m*_*M*_ the maintenance energy rate per motor volume (moles of ATP consumed/volume of motor/unit of time), *e*_*P*_ the energy demand of protein synthesis (molecules of ATP consumed per amino acid), *f*_*C*_ the carbon uptake (moles carbon/cell volume/time), *f*_*E*_ and *f*_*M*_ the energy production and maintenance demand (moles ATP/cell volume/unit of time), *γ*_*F*_ and *γ*_*O*_ the ATP yields per carbon of fermentation and oxidative phosphorylation, and *γ*_*aa*_ is the average carbon content per amino acid. Finally *Y* quantifies the yield of carbon incorporation into biomass.

The equations (4) and (5) encode the proper bookkeeping of the cell volume fractions and protein concentrations. The equations (6), (8) and (9) encode the metabolic balance between production and consumption of carbon, energy and proteins, respectively. The model analysis is simplified by lumping together the energy producing pathways. Introducing the notation *ϕ*_*F*_ = *ωϕ*_*E*_ and *ϕ*_*O*_ = (1 − *ω*)*ϕ*_*E*_, where *ϕ*_*E*_ is the total volume fraction occupied by energy producing pathways and *ω* the fraction of *ϕ*_*E*_ accounted by fermentation enzymes, equation (8) can be rearranged to obtain

11 $${\varphi }_{E}=\frac{{m}_{M}}{{h}_{E}}{\varphi }_{M}+\frac{{e}_{P}{h}_{R}}{{h}_{E}}{\varphi }_{R}$$

where

12 $${h}_{E}=\omega {h}_{F}+\mathrm{(1}-\omega ){h}_{O}$$

Substituting *ϕ*_*E*_ and *ϕ*_*M*_ in equation (4) by their expressions in equations (11) and (3), we arrive to the following equation for the ribosomal volume fraction

13 $${\varphi }_{R}=\frac{1}{1+\frac{{e}_{P}{h}_{R}}{{h}_{E}}}[\varphi -{\varphi }_{0}-(1+\frac{{m}_{M}}{{h}_{E}})\frac{{V}_{M}}{{V}_{c}}\frac{{k}_{{\rm{B}}}T}{{E}_{M}}\,\mathrm{ln}\,\frac{{\rm{\Phi }}}{{\rm{\Phi }}-\varphi }]$$

Substituting *ϕ*_*E*_ and *ϕ*_*R*_ in equation (5) by (11) and (13), respectively, we obtain

14 $$\frac{1}{\mu +{k}_{P}}=\frac{1}{{\mu }_{B}}+\frac{1}{{\mu }_{C}}$$

with the definitions

15 $${\mu }_{B}=\frac{{h}_{R}}{{c}_{R}}\frac{1}{1+\frac{{e}_{P}{\nu }_{R}}{{\nu }_{E}}}$$

16 $${\mu }_{C}=\frac{{h}_{R}{\varphi }_{R}}{{C}_{ng}}$$

where *ν*_*R*_ = *h*_*R*_/*c*_*R*_ is the ribosome translation rate divided by the amino acid content of a ribosome, *ν*_*E*_ = *h*_*E*_/(*ωc*_*F*_ + (1 − *ω*)*c*_*O*_) is the energy production rate divided by the amino acid content in proteins associated with energy production, and

17 $${C}_{ng}={c}_{0}{\varphi }_{0}+({c}_{M}+{c}_{E}\frac{{m}_{M}}{{h}_{E}})\frac{{V}_{M}}{{V}_{c}}\frac{{k}_{{\rm{B}}}T}{{E}_{M}}\,\mathrm{ln}\,\frac{{\rm{\Phi }}}{{\rm{\Phi }}-\varphi }$$

Finally, *C* and *f*_*C*_ can be expressed as a function of *ϕ*_*R*_, *ϕ*_*E*_ and *μ* using equations (6) and (9), obtaining

18 $$C=\frac{{h}_{R}{\varphi }_{R}}{{k}_{P}+\mu }$$

19 $${f}_{C}=(\omega \frac{{h}_{F}}{{\gamma }_{F}}+\mathrm{(1}-\omega )\frac{{h}_{O}}{{\gamma }_{O}}){\varphi }_{E}+\frac{\mu }{{k}_{P}+\mu }\frac{{h}_{R}}{{\gamma }_{aa}}{\varphi }_{R}$$

The equations above are analytical expressions allowing us to investigate the model behavior in detail, including the dependencies on model parameters. Equation (14) indicates that the growth rate is the harmonic mean between the biosynthetic and molecular crowding growth rates *μ*_*B*_ and *μ*_*C*_, respectively. The biosynthetic growth rate (*μ*_*B*_, equation 15) is proportional to the ribosome efficiency *h*_*R*_/*c*_*R*_ times a correction accounting for the biosynthetic cost of energy generating enzymes. It tells us that cells cannot grow faster than the speed at which ribosomes synthesize ribosomal protein. We note that *μ*_*B*_ is independent of *ϕ* and *ϕ*_0_. It is only a function of *ω* and, therefore, it only depends on the relative contribution of fermentation and oxidative phosphorylation to energy generation. The molecular crowding growth rate (*μ*_*C*_, equation 16) is simply given by the ratio between the protein synthesis rate and the growth-independent component of the protein content (*C*_*ng*_, equation 17). It tell us that cells cannot growth faster than the speed at which ribosomes synthesize non-growth related proteins. In contrast, we do not expect the model predictions to be highly accurate. This is a very simplified model of cell metabolism built for the purpose of understanding key features. In the following sections we analyze some of the model achievements.

### Osmolarity dependency of the growth rate

The growth rate defined by (14) is an implicit function of *ϕ*, through the *ϕ* dependencies of the ribosome volume fraction *ϕ*_*R*_ (13) and the protein concentration not related to growth *C*_*ng*_ (17). *ϕ*_*R*_ is a concave function of *ϕ* (13), with a maximum at the volume fraction

20 $${\varphi }^{\ast }={\rm{\Phi }}-(1+\frac{{m}_{M}}{{h}_{E}})\frac{{V}_{M}}{{V}_{c}}\frac{{k}_{{\rm{B}}}T}{{E}_{M}}$$

**Figure 3:**
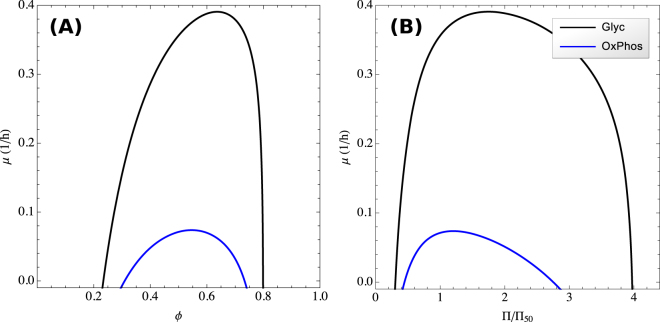
Osmolarity dependency of the growth rate. (**A**) Maximum growth rate as a function of the excluded volume fraction, when energy is produced by fermentation (black line) or oxidative phosphorylation (blue line). (**B**) Maximum growth rate as a function of external osmolarity. These plots were obtained using parameters for mammalian cells.

Since *C*_*ng*_ is an increasing function of *ϕ* and *μ*_*C*_ and *μ* in equations (16) and (14) are also concave functions of *ϕ*, they have a maximum in the range 0 < *ϕ* < ϕ^*^. Figure 3A shows the dependency of the growth rate with the excluded volume fraction *ϕ*, for the case when energy is produced by pure oxidative phosphorylation (*ω* = 0, blue line) or fermentation (*ω* = 1, black line). This plot corroborates that the growth rate attains a maximum for an intermediate volume fraction. Furthermore, at any given *ϕ* the fermentation line exhibits a higher value, indicating that fermentation can sustain higher growth rates than oxidative phosphorylation. This is explained by the fact that fermentation has a higher horsepower (*h*_*F*_ > *h*_*O*_), *i.e*. it can produce more energy per volume of enzyme than oxidative phosphorylation.

We also note that the plot of *μ* vs. *ϕ* follows a bell like shape. A similar behavior has been observed for the growth rate dependency with medium osmolarity^20–25^. The ideal osmotic response of cells from a reference state (^*^) to a new external osmolarity follows the Boyle–van’t Hoff relation Π(*V* − *b*) = Π^*^(V^*^ − *b*)^26^, where Π^*^ and Π are the osmotic pressures at the reference and new states, V^*^ and *V* are the corresponding cell volumes, and *b* is the solvent excluded volume occupied by macromolecules. *b* is assumed to be constant between the two conditions as observed experimentally. The Boyle–van’t Hoff relation can be rewritten to obtain a relationship between *ϕ* = *b*/*V* and the osmotic pressure, *ϕ* = Π/(Π_50_ + Π), where Π_50_ = Π^*^ϕ^*^/(1 − ϕ^*^) is the osmotic pressure where macromolecules occupy 50% of the cell volume. Substituting the later equation into the growth rate equation (14) we can plot the growth rate as a function of the relative osmotic pressure Π/Π_50_ (Fig. 3B). The resulting behavior explains the observed bell shape plot of growth rate vs. osmolarity.

### Energy scales of cell metabolism

**Figure 4:**
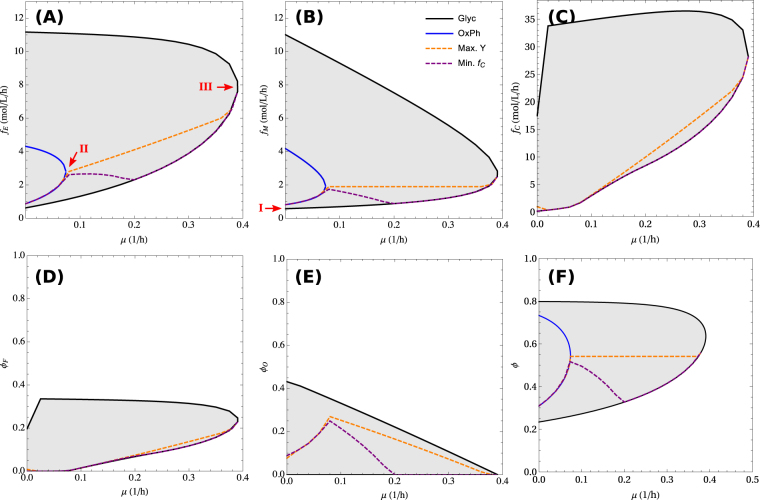
Energy balance as a function of growth rate. (**A,B**) Energy demand of all metabolism (**A**) and cell maintenance (**B**) as a function of the growth rate. The arrows indicate the three energy scales of metabolism: I. maintenance, II. switch to fermentation, and III. maximum growth rate. (**C**) Carbon consumption. (**D**,**E**,**F**) Volume fractions of fermentation enzymes (**D**), oxidative phosphorylation machinery (**E**) and biomass (**F**) as a function of growth rate. The shaded area represents the range of feasible values corresponding to different values of *ϕ*. The blue curve is the maximum growth rate obtainable with pure oxidative phosphorylation. The dashed lines show the trajectories corresponding to lowest carbon consumption (purple) and maximum growth yield (orange). These plots were obtained using parameters for mammalian cells.

At a given growth rate, the total energy demand and maintenance energy demand can vary significantly depending of the excluded volume fraction (Fig. 4A,B). However, solutions that are further constrained to satisfy optimality criteria are concentrated on the lower *ϕ* range of the feasible space. We obtained solutions corresponding to maximum yield (orange dashed line, see equation 10) and minimal carbon uptake (purple dashed, see equation 19), computed by a constrained optimization routine in Mathematica. As expected the overall energy demand increases with increasing the growth rate for the optimal solutions (Fig. 4A). In contrast, it is not clear *a priori* whether the maintenance energy demand changes or not with the growth rate. From equation (3) it follows that solutions where *ϕ* is constrained to be a constant are characterized by a constant *ϕ*_*M*_ and, therefore, a growth independent maintenance energy demand. We also take a closer look to the maintenance energy demand for the solution maximizing growth yield (Fig. 4B, orange line). In this case, the maintenance energy demand remains approximately constant relative to the total energy demand, except for the extreme values close to zero and maximum growth rate. This implies that the energy of cell maintenance may appear to be independent of the growth rate even though we have not imposed such a constraint. The motors volume fraction associated with this constant energy demand is *ϕ*_*M*_ = *f*_*E*_/*m*_*M*_ ≈ 0.05. We have also estimated the molecular motors fraction from proteomic data reported for different cell lines (Methods). The estimates are in the range of 0.02 to 0.08, in agreement with what is expected from the model.

An important question in cell metabolism is why cells exhibit active fermentation even under conditions where oxygen is present and oxidative phosphorylation should be the pathway of choice. This problem can be addressed within our model by analyzing its solution when both fermentation and oxidative phosphorylation can be active. Because of their redundant nature for energy generation, the volume fractions occupied by the oxidative phosphorylation and fermentation machineries vary widely (Fig. 4C,D). Since the maximum growth rate sustained by oxidative phosphorylation is smaller than that sustained by fermentation, it is evident that there is a threshold growth rate above which fermentation is obligatory (Fig. 4C). In compensation the maximum rate of oxidative phosphorylation goes down with increasing proliferation rate (Fig. 4D). The later behavior is in agreement with the observed decrease of mitochondria protein mass in yeast^25^ and mammalian cells^6^ at higher growth rates.

Taking all this evidence together we provide an explanation for the three key energy scales of cell metabolism. From the quantitative point of view we focus on values for mammalian cells. The maintenance energy demand is represented by the energy expend by molecular motors. Provided that the motors persistence parameter *p* is of the order of 1, the energy demand of cell maintenance predicted from this model (~0.5 mol/L/h, Fig. 4B,I) is in the range of what is observed experimentally (around 0.3 mol ATP/L/h)^3^. This is a striking observation given that our estimate is based on microscopic parameters characterizing molecular motors (Methods). The energy demarking the switch to obligatory fermentation is simply a consequence of fermentation having a larger horsepower than oxidative phosphorylation. This results in an energy demand to switch to obligatory fermentation of ~3 mol/L/h (Fig. 4A,II), within the range of experimental observations (~2 mol ATP/L/h)^6^. Finally, the energy demand at maximum growth rate is simply determined by the energy demand at the maximum growth rate that can be achieved using fermentation (~8 mol/L/h, Fig. 4A,III), again in very good agreement with the maximum fermentation rates reported for mammalian cells (~8 mol ATP/L/h)^6^.

The maximum yield and minimum carbon consumption solutions are similar in that they tend to avoid fermentation as much as allowed by the physical constraints (Fig. 4D). Indeed, fermentation results in carbon excretion and increases carbon consumption due its lower energy yield. However, these two solutions are different because maximum growth yield implies maximum incorporation of carbon into biomass, which does not necessarily imply minimum carbon consumption. In particular, the maximum yield solution results in higher volume fractions for the oxidative phosphorylation pathway (Fig. 4E), concomitantly with a higher maintenance energy demand associated with the molecular motors activity (Fig. 4B). These changes result in a higher volume fraction (Fig. 4F) and higher carbon consumption (Fig. 4C) than the minimum carbon uptake solution.

### Background proteins reduce the metabolic capacity

**Figure 5:**
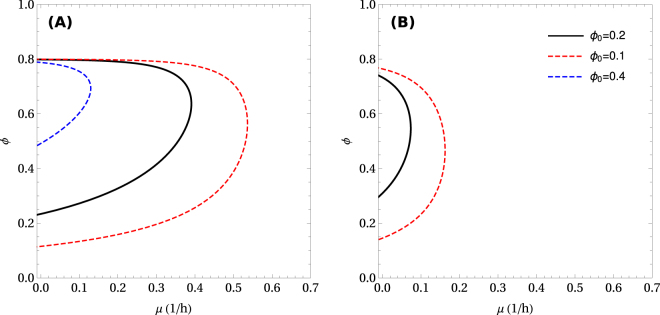
Impact of the volume fraction of background proteins. Growth rate vs. *ϕ* for different values of *ϕ*_0_. (**A**) Pure fermentation, (**B**) pure oxidative phosphorylation.

Another important observation is the dependency of the model solution with the volume fraction of background proteins *ϕ*_0_. The solutions described above were obtained assuming a typical value of *ϕ*_0_ = 0.2 as observed for mammalian cells (Methods). However, changing *ϕ*_0_ can have a big quantitative impact on the model solution. Reducing *ϕ*_0_ by half we obtain a significant increase in the maximum growth rate and the range of *ϕ* with feasible solutions (Fig. 5A). In contrast, doubling *ϕ*_0_ reduces the maximum growth rate and constraints the solutions to a smaller *ϕ* range concentrated near the maximum packing density (Fig. 5A). For a healthy organism, we think there is not much flexibility on the *ϕ*_0_ values. First, the background protein content is constrained by the actin cytoskeleton that is required to support the molecular motors activity. Just to get an order of magnitude, the rate of ATP hydrolysis by myosin is characterized by a half saturation constant for actin of the order of 40 *μ*M^27,28^. That value multiplied by a typical molar mass of 500 kDa and a specific excluded volume of 2 mL/g yields a volume fraction of 0.04. On top of that we need to add the volume of the nuclei (eukaryotes) or nucleoid regions (prokaryotes). The nucleus occupies a volume fraction of 0.06 to 0.1 in mammalian cells^29–31^, bringing the lower bound of *ϕ*_0_ to about 0.15. Third, there are other metabolic enzymes associated with lipid and nucleotide metabolism that can occupy a significant volume fraction. For example, fatty acid synthase alone occupies a typical volume fraction of 0.006 (Methods). Therefore, a value of *ϕ*_0_ = 0.2 is about what we expect for mammalian cells. However, *ϕ*_0_ takes different values in other organisms. For yeast cells *ϕ*_0_ ≈ 0.1, and that may explain why yeast cells can grow faster than mammalian cells (Fig. 5A, *ϕ*_0_ = 0.1 vs 0.2). It is also worth noticing that *ϕ*_0_ could increase above 0.2 in pathological conditions where protein aggregation occurs. In such instances there can be a dramatic reduction in the metabolic capabilities of cells (Fig. 5A, *ϕ*_0_ = 0.4 vs 0.2). The results obtained if cells are forced to rely on pure oxidative phosphorylation are shown in Fig. 5B. Note that in this case the value *ϕ*_0_ = 0.4 becomes infeasible, implying that higher levels of *ϕ*_0_ force a switch to obligatory fermentation. To better understand these effects, note that from eq. (14), increasing *ϕ*_0_ at constant *ϕ* is balanced by a decrease in *ϕ*_*g*_, the portion of intracellular space allocated to growth-related functions, and an increase in *C*_*ng*_, the proteomic mass alloted to non-growth associated activities.

### Temperature dependency of the growth rate

**Figure 6:**
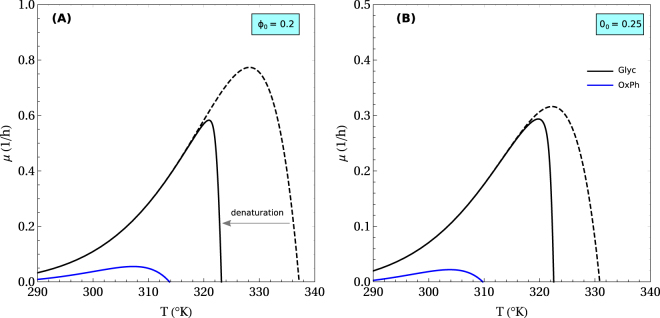
Temperature dependency of the maximum growth rate. This plot was obtained using parameters for mammalian cells. Dashed lines are the prediction obtained without considering protein denaturation. Continuous line considers protein denaturation^33^. The blue curve is the maximum growth rate by pure oxidative phosphorylation. (**A**) For a background protein fraction *ϕ*_0_ = 0.2, and (**B**) for a background protein fraction *ϕ*_0_ = 0.25.

The molecular motors fraction that is required to counteract molecular crowding is proportional to the temperature (eq. 3). This implies that the cost associated with molecular motors is higher with increasing temperature and should result in a drop of metabolic rate at high temperatures. That together with the Arrhenius-like increase of enzyme rates with temperature, ${e}^{-{E}_{a}/{k}_{{\rm{B}}}T}$, would result in a maximum growth rate at some intermediate temperature. Figure 6A shows the temperature dependence of the growth rate, after accounting for the Arrhenius temperature dependencies of the horsepower of ribosomes and fermentation and the kicking rate of molecular motors (dashed). The qualitative shape of the curve is strikingly similar to what is observed experimentally^32^. The precise temperature where the maximum growth rate is achieved is in part determined by the actual values of the activation energies. It is quite impressive that this simple model can already reproduce the temperature dependency of the growth rate without taking into consideration the propensity of proteins to denature at high temperatures. To investigate the impact of protein denaturation at higher temperature, we used the Ghosh-Dill formula^33,34^ and an assumed protein length distribution of the Gamma form^35^ (Methods). Protein denaturation results in an increase in *ϕ*_0_ by the contribution of unfolded proteins. This shifts the optimal growth rate, and the cell-death temperature to lower values, consistent with experimental values of mesophiles^36^.

The combination of increased *ϕ*_*M*_ and *ϕ*_0_ with increasing temperature will carry as a consequence a reduction of the cell volume fraction available to the metabolic machinery. Therefore, following the analysis of previous sections, there would be a temperature above which pure oxidative phosphorylation cannot sustain the cell energy demand. The blue line in Fig. 6A shows the maximum growth rate attainable by oxidative phosphorylation. Above this line fermentation is obligatory. Based on this plot, and the corresponding choice of model parameters, fermentation becomes obligatory above ~310 K. Above this temperature pure oxidative phosphorylation cannot even sustain the energy demand of cell maintenance of non-growing cells (*μ* = 0). This is an interesting observation, indicating that overflow metabolism can be also induced by increasing temperature. Finally, as expected, cells with higher content of background proteins are more sensitive to temperature changes and the lines are shifted to lower temperatures and growth rates (Fig. 6B).

## Discussion

We have investigated a physical model of cell metabolism where the energy demand of cell maintenance represents the energy expended by molecular motors to counteract the entropic forces associated with molecular crowding. Our model considers, in a simplified manner, the autocatalytic nature of cell growth^10,37^, the volumetric and biosynthetic costs of metabolic enzymes, ribosomes and other components^5^, and the existence of alternative metabolic pathways for the degradation of substrates (fermentation vs. oxidative phosphorylation)^5^. This represents an improvement over the “enzyme allocation” models that have appeared over the last decade to explain overflow metabolism^3,6,9–11,15,37–39^. The new addition is a mechanistic model for the energy demand of cell maintenance, which is included as an empirical constant in previous approaches. Our model predicts an energy demand for cell maintenance that is in the range of what is observed experimentally. More importantly, the model exhibits a rich behavior depending on the macromolecular volume fraction and growth rate. It predicts the growth rate threshold for the metabolic switch to obligatory fermentation and the maximum growth rate. The model also explains the bell shape curve of growth rate as a function of osmolarity and temperature.

Our theory is built upon the observation that ATP dependent vibrations fluidize the cytoplasm^18,19,40^, but is independent of molecular mechanisms driving these motions. In eukaryotic cells, kinesins, myosins and dyneins exert forces on the order of pico Newtons on other molecules^41^. Based on current knowledge these molecular motors are not present in prokaryote cells. Yet, ATP dependent motion has also been observed in bacterial cells. Chromosomal loci in *E. coli* jiggle in a manner that is sensitive to metabolic activity and cannot be explained by thermal motions alone^42^. RNA polymerase contributes to the observed motion, but additional (as yet unknown) molecular entities are required to explain the full extent of the vibrations. Moreover, cytoplasmic diffusion of fluorescent proteins in *E. coli* is highly dependent on ATP concentrations and thus non-thermal^18^. This suggests that our model might apply to prokaryotic cells too.

There are several predictions that will require further testing for verification. The model predicts that the maintenance energy demand is not necessarily independent of the growth rate. Therefore we cannot assume a linear extrapolation of the energy demand to zero growth rate. Based on our analysis this would be valid only if cells operate at constant macromolecular volume fraction. In this regard, the maximum yield solution of the model implies a constant macromolecular volume fraction in the phase with mixed oxidative phosphorylation (Fig. 4F). Since the protein density is constant in this regime, we can work indistinctively with a volume or protein fraction allocation constraint.

The temperature dependence of the growth rate of microbes is typically modelled under the assumption that there is a core group of rate limiting proteins that control growth^33,36,43^. Therefore these models require knowledge of this group of essential proteins. This assumption is unnecessary in our model. Rather, growth rate decreases at higher temperatures by a combination of two factors. First, higher temperatures imply higher costs associated with molecular motors. Second, the accumulation of denatured proteins leads to a build-up of *ϕ*_0_, reducing the space available for functional ribosomes and metabolic components contributing to growth. Moreover, cells having a higher basal share of background proteins are more sensitive to temperature variations. Cancers cells have been reported to be more sensitive to high temperatures than normal cells^44,45^. A plausible explanation consistent with our model is that cancer cells have a larger set of background of proteins (*e.g*. defective proteins due to mutations). Further work is required to understand additional effects, such as the heat shock response. In a recent work^46^, a detailed genome-scale model of *E. coli* was developed that includes chaperone mediated protein folding and explains the temperature dependence of growth in this organism. This model requires data of sequences and structures of enzymes, folding kinetic rates, folding equilibrium constants, thermo-stabilities and aggregation propensities. Most of these data is not available for other organisms. Moreover, although full-scale detailed models are valuable to make predictions that are as complete and accurate as possible, it is hard to interpret simulation results due to their complexity.

It is also evident that a metabolic switch to obligatory fermentation can be obtained in many ways. We have discussed the metabolic switch with increasing growth rate. The same behavior can be achieved by increasing the rate of protein turnover, effectively modeling the impact of protein secretion on cell metabolism. That may explain why non-growing fibroblasts with high rates of protein secretion exhibit high rates of fermentation^47^. A metabolic switch to obligatory fermentation could be also achieved at constant growth rate and going from low to high macromolecular excluded volume fraction. Moreover, the growth rate at which the switch occurs is predicted to be temperature dependent. The currently accepted theory^3,5,11^ is that enzymatic costs force the cell to switch to fermentation at higher growth rates. We have extended this picture to include the effects of osmolality^48^ and temperature.

Finally, the model introduced here can be extended to more comprehensive representations of cell metabolism. To this end we can introduce the more general formulation of metabolic balance *Sf* = 0, with *f* representing the vector of steady state reaction rates and *S* the stoichiometric matrix of the metabolic network. These metabolic fluxes would be further constrained by the reaction rate capacities *f*_*i*_ ≤ *h*_*i*_*ϕ*_*i*_, with *h*_*i*_ representing reaction horsepowers and *ϕ*_*i*_ enzyme volume fractions. This generalization will allow us to understand the impact of molecular crowding and the counteracting motors activity on metabolic pathways beyond energy and protein metabolism.

## Methods

### Calculation of entropic pressure of molecular crowding

To derive Equation (1) in the main text, we consider a regular lattice of *N* spots where *n* hard spheres can be placed. A maximum of one sphere is allowed per spot. The configurational entropic cost associated with the creation of a hole of size equal to *m* spots is given by:

21 $$\mathrm{ln}(\begin{array}{c}N\\ n\end{array})-\,\mathrm{ln}(\begin{array}{c}N-m\\ n\end{array})\approx ({H}_{N}-{H}_{N-n})m\approx -\,m\,\mathrm{ln}(\frac{N-n}{N})$$

where ${H}_{k}={\sum }_{i=1}^{k}\mathrm{1/}i$ are the Harmonic numbers. In the first approximation we employed a Taylor expansion of the function $$\mathrm{ln}(\begin{array}{c}x\\ n\end{array})$$ about *x* = *N* to first order, and in the second we approximated *H*_*k*_ ≈ ln*k*. Both approximations are accurate under the assumptions $N,n\gg m$, $N\gg 1$, $N-n\gg 1$. It follows that:

22 $$\frac{1}{{V}_{h}}[\mathrm{ln}(\begin{array}{c}N\\ n\end{array})-\,\mathrm{ln}(\begin{array}{c}N-m\\ n\end{array})]\approx -\frac{1}{{V}_{c}}\,\mathrm{ln}(\frac{{\rm{\Phi }}-\varphi }{{\rm{\Phi }}})$$

where *V*_*c*_ is the typical volume of macromolecular crowders, *V*_*h*_ = *mV*_*c*_ the hole volume, *ϕ* = *nV*_*c*_/*V* the fraction of cellular volume occupied by macromolecular crowders, Φ = *NV*_*c*_/*V* the maximum volume fraction available for macromolecular crowders, and *V* the cell volume.

### Calculation of the molecular motors pressure

Consider an homogeneous gas of molecular motors on a cubic chamber of size *L* as a simplified model of a hole surrounded by macromolecules. Each motor has a kicking rate *κ*. During a kick, the motor exerts a force *F* for a duration *τ* ≤ 1/*κ* and moves a distance *d*. Moreover, each motor has an orientation (that we assume is isotropically distributed) which determines the direction of the applied force and of the step. For each motor, let *ξ* be the projection of the motor orientation onto the normal of the wall (−1 ≤ *ξ* ≤ 1), and let *x* be its distance to the wall. If this motor kicks, it will reach a given wall if *x* ≤ *dξ*, in which case it will transfer a momentum:

23 $$\tau F\xi (1-\frac{x}{d\xi })$$

since the projection of the force against the wall is *Fξ*, and a fraction *x*/(*dξ*) of the kick duration *τ* is spent reaching the wall. Here *x* and *ξ* are uniformly distributed random variables, with −1 ≤ *ξ* ≤ 1 and 0 ≤ *x* ≤ *L*. The average impulse transferred per kicking particle can be obtained taking into account that the probability distribution of the projection of a random unit vector in three-dimensions onto an axis is uniform, resulting in

24 $$I={\int }_{0}^{L}\frac{{\rm{d}}x}{L}{\int }_{-1}^{1}\frac{{\rm{d}}\xi }{2}\tau F\xi (1-\frac{x}{d\xi }){\rm{\Theta }}(d\xi -x)=\frac{\tau Fd}{12L}$$

where Θ(*x*) is Heaviside’s theta function. This of course assumes that *d* < *L*. The pressure applied to the wall is determined by the impulse per motor *I*, times the kicking rate *κ*, times the number of motors in the chamber *n*_*M*_, divided by the wall area *L*^2^

25 $$P=\frac{{n}_{M}I\kappa }{{L}^{2}}=\frac{{n}_{M}}{V}\frac{pFd}{12}$$

where *p* = *τκ*. The motors can perform more than one kick in the same direction once they get in contact with a macromolecule. In such a case *p* would be redefined as *p* = *rτκ* where *r* > 1 is the average number of kicks upon contact with a macromolecule. Since *τκ* ≤ 1 and *r* ≥ 1 the parameter *p* is expected to be of the order of 1.

### Protein denaturation

The fraction of denatured proteins at temperature *T* is estimated as $\sigma (T)={\int }_{0}^{\infty }\frac{P(N)}{1+{e}^{-{\rm{\Delta }}G(N,T)/(RT)}}{\rm{d}}N$, where Δ*G*(*N*, *T*) is the average folding free energy of proteins of length *N* and *P*(*N*) is the frequency of proteins of length *N*. The folding free energy is estimated using the Ghosh-Dill formula for mesophiles (Eq. 1 of ref.^33^). The protein length distribution is assumed to be of the Gamma form with parameters reported for yeast^35^. Finally, the fraction of background proteins *ϕ*_0_ increases by *σϕ*, representing unfolded proteins.

### Parameter estimation

The model depends on a number of parameters characterizing the activity of motor proteins and metabolic enzymes which are described here. In the supplementary text we discuss sensitivity to parameter values in more detail (Figures S1–S5) and a summarized parameter table is given (Table S1).

#### Proteins specific excluded volume

The average specific excluded volume of macromolecules in cells is *v*_*s*_ = 2 mL/g for proteins (Figure A2c in ref.^49^). Since cells are made mostly made of proteins, 2 mL/g can be taken as an estimate of the typical specific excluded volume of proteins in cells. An independent estimate has been obtained by electrospray ionization mass spectrometry, which results in an average effective protein density of 0.58 g/mL^50^, or equivalently a specific excluded volume of 1.7 mL/g, close to the 2 mL/g value reported for cell extracts.

#### Volume fraction of fatty acid synthase

Using publicly available data^51^, we calculated an average mass fraction of fatty acid synthase in the proteome of the NCI60 panel of cancer cell lines of 0.02. Multiplying by the average protein density of 0.15 g/mL^52^ and by the protein excluded volume, 2 mL/g, yields the estimated volume fraction of 0.006.

#### Volume fraction of background proteins

Following the same procedure as in the estimation of the volume fraction of fatty acid synthase, we estimated the volumetric fraction of background proteins by computing the fraction left after excluding fermentation enzymes, molecular motors, mitochondrial and ribosome proteins. Protein localization and function were distinguished following annotations from the Gene Ontology database^53^. The estimated volume fraction occupied by background proteins varies from 0.1 up to 0.4 in the panel of NCI60 cell lines^51,52^, with an average value of 0.2.

#### Compartmental protein concentrations

The compartmental protein concentrations of fermentation, motors and background proteins was calculated using *c*_*i*_ = 1/(*m*_*a*_*v*_*s*_) = 4.6 mol/L, where *i* = *F*, *M*, 0, *m*_*a*_ = 109 g/mol is the average molar mass of amino acids and *v*_*s*_ = 2 mL/g is the protein specific excluded volume. Using the eukaryotic ribosome volume of 4000 nm^3^ and a composition of 11590 amino acids/ribosome^54,55^, we calculated *c*_*R*_ = 4.8 mol/L. The specific volume of mitochondria in mammalian cells is ${v}_{mito} \sim 2.6$ mL of mitochondria/g of mitochondrial protein. Then *c*_*O*_ = 1/(*m*_*a*_*v*_*mito*_) ≈ 3.5 mol/*L*.

#### Fermentation horsepower

Reference^56^ reports the rate of fermentation between 0 and 40 °C from an *in vitro* reconstitution of glycolysis enzymes with 40 mg/mL of total glycolytic protein concentration. This data is well fitted to the Arrhenius law of the rate of a biochemical reaction ${f}_{F}={f}_{0}{e}^{-{E}_{a}/RT}$, with *f*_0_ = 7.5×10^13^ *μ*mol/min/mL and *E*_*a*_ = 72.6 kJ/mol (Supplementary Materials, Figure S5). Using this Arrhenius law we obtain the fermentation rate at 37 °C to be 45 *μ*mol/min/mL. Dividing by a protein excluded volume fraction of 40 mg/mL × 2 mL/g = 0.08 we obtain a fermentation horsepower of *h*_*F*_ = 34 mol ATP/L/h. A theoretical estimate can be obtained under the assumption that all enzymatic steps of glycolysis are at saturation, resulting in ${h}_{F}=\mathrm{1/(}{v}_{s}{\sum }_{i}({s}_{i}/{k}_{i}))$, where *v*_*s*_ is the proteins specific excluded volume, the sum run over all glycolysis enzymes, *s*_*i*_ is the stoichiometric coefficient of reaction *i* relative to lactate release and *k*_*i*_ is the specific turnover rate of the enzyme catalyzing step *i*. Based on specific turnover data from BRENDA^57^, *k* = 36.5, 620, 90, 22.3, 8000, 68.1, 600, 1.5, 70, 400, and 106 *μ*mol/mg/min for the enzymes hexokinase, phosphoglucose isomerase, phosphofructose kinase, alodolase, glyceraldehyde-3-phosphate dehydrogenase, phosphoglycerate kinase, phosphoglycerate mutase, enolase, pyruvate kinase and lactate dehydrogenase, respectively. Furthermore, *s*_*i*_ = 1/2 from the hexokinase to the aldolase step and *s*_*i*_ = 1 from glyceraldehyde-3-phosphate dehydrogenase to lactate dehydrogenase. Using these enzyme specific turnovers, stoichiometric coefficients, and a protein specific excluded volume of 2 mL/g we obtain *h*_*F*_ = 40 mol ATP/L/h. This theoretical estimate is in excellent agreement with the experimentally measured value. The calculations and figures reported in the main text were obtained using the experimental value of *h*_*F*_ = 34 mol ATP/L/h.

#### Oxidative phosphorylation horsepower

The oxidative phosphorylation horsepower in mammalian cells can be estimated from experimental reports of the maximum capacity for ATP production by isolated mitochondria^5^. The average value is *h*_*O*_ = 10 ATP mol/L of mitochondria/h for mitochondria isolated from healthy mammalian cells. For mitochondria isolated from cancer cells it goes down to 3 mol ATP/L of mitochondria/h. An independent theoretical estimate was obtained using a mathematical model of mitochondrial oxidative phosphorylation^58^. Constraining the total enzymatic mass of oxidative phosphorylation and then maximizing the rate of ATP production, we obtain a theoretical oxidative phosphorylation horsepower of 19 mol ATP/L of mitochondria/h. This value closely matches the maximum mitochondrial horsepower reported across multiple cell types^5^. The calculations and figures reported in the main text were obtained using the average experimental report for healthy mitochondria of *h*_*O*_ = 10 ATP mol/L of mitochondria/h.

#### Ribosomes horsepower

The horsepower of mammalian ribosomes is *h*_*R*_ = 8.4 mol of amino acid incorporated/L of ribosome/h^5^.

#### Motors parameters

Molecular motors exert forces of the order of *F* = 5 pN per kick moving for about *d* = 10 nm^41,59^. The motors kicking rate is $\sim \kappa =5\,{s}^{-1}$, while the duration of a kick is $\tau  \sim 0.5$ s^30,60^. This results in a persistence parameter, *p* = *rκτ*, where *r* is the number of repeated kicks upon contact with a macromolecule. The average repeated kicks *r* ≈ 5 was estimated so as to match a maintenance demand of ≈1 mol/L/h at a typical macromolecular volume fraction of *ϕ* = 0.4^49^. The motors maintenance energy per motor volume was calculated as *m*_*M*_ = *κ*/(*M*_*M*_*v*_*s*_), where *M*_*M*_ is the motors molar mass and *v*_*s*_ is the protein specific excluded volume. We use the molar mass of Myosin Va, *M*_*M*_ = 215405 g/mol (UNIPROT:Q9Y4I1) as a typical value. This results in *m*_*M*_ = 42 mol/h/L.

#### Molecular motors volume fraction

Using publicly available data^51^, we estimated the mass fraction of molecular motors to be between 0.07 and 0.27. We employed the gene ontology^53^ terms GO:0003774 (motor activity) and GO:0016887 (ATPase activity) to define the set of molecular motor proteins. Multiplying by the average protein density of 0.15 g/mL^52^ and by the protein excluded volume, 2 mL/g, yields the estimated volume fraction range 0.02 to 0.08.

#### Activation energies

The biochemical horsepowers were assumed to follow an Arrhenius dependence on the temperature, ${h}_{i}={h}_{i0}{e}^{\frac{{E}_{i}}{R{T}_{0}}-\frac{{E}_{i}}{RT}},$where *i* = *R*, *F*, *T*_0_ is the reference temperature where the horsepower equals *h*_*i*0_ and *E*_*i*_ is the activation energy. Since the motors kicking is also an activated process, the maintenance requirement of motors was assumed to follow a similar law, ${m}_{E}={m}_{E0}{e}^{\frac{{E}_{M}}{R{T}_{0}}-\frac{{E}_{M}}{RT}}$. The activation energies were estimated from the literature: ribosomes 80 kJ/mol^61^, glycolysis 77 kJ/mol^56^, and myosin 125 kJ/mol^62^. The activation energy of oxidative phosphorylation was estimated from that of ATP synthase in yeast 50 kJ/mol^63^.

### Data Availability

All data required to perform the calculations can be found in the main text.

## Electronic supplementary material

Supplementary text
