# Supplementary material for: A physical model of cell metabolism
Source: Sci Rep. 2018 May 29;8:8349. doi: 10.1038/s41598-018-26724-7 (PMC5974398; doi:10.1038/s41598-018-26724-7)
Supplement: Supplementary file 1 — Supplementary text [file 41598_2018_26724_MOESM1_ESM.pdf]

A physical model of cell metabolism. Supplementary Text

Jorge Fernandez-de-Cossio-Diaz and Alexei Vazquez

March 20, 2018

Sensitivity of model results to parameter values

To study the sensitivity of the model to variations in parameter values, we focused on parameters with the largest uncertainty or expected to exhibit larger variations between cell types. Since the molecular mechanisms behind non-thermal motion of macromolecules in bacteria have not been elucidated [1, 2], we focus on parameters that have been measured for eukaryotic cells and where it is possible to assess their range of variation.

The composition parameters c_F, c_M, c_0, c_R, c_O, and the horsepowers h_F, h_R, depend on structural properties that are largely conserved between many cell types, and therefore are not expected to vary significantly. Therefore we focus our analysis on the mitochondrial horsepower (h_O), parameters of molecular motors (F, d, p, V_M), crowder volume (V_c), and fraction of background proteins (ϕ_0).

h_O varies between 2 and 20 mol ATP/h/L in multiple cell types, including yeast, cancer and healthy human cells [3]. In the model we used the value 10 mol ATP/h/L, characteristic of healthy human tissues. Interestingly the lowest values of h_O are exhibited by cancer cells. Figure S1 shows the effect of varying this parameter between 8 and 20 mol ATP/h/L (cf. Figures 4A,B in the main text). The difference is only quantitative. Moreover, the value of h_O has no impact on the energy demand of maximum growth, since this regime is characterized by a switch to obligatory fermentation. Decreasing h_O only affects the growth rate of the pure OxPhos regime. At values lower than 8 mol ATP/h/L, pure oxidative phosphorylation (OxPhos) becomes unfeasible in our model, indicating that these cells must ferment even in a quiescent state.

The parameters characterizing molecular motors affect the model only through the non-dimensional ratio $\theta = \frac{V_M}{V_c} \frac{1}{pFd}$, which determines the fraction of molecular

motors in the cell biomass. In the calculations in the main text, we obtained the value $\theta = 0.04$ (see Methods). It is hard to assess the extent of variation of this parameter from the scarce experimental data available. To assess the impact of its variation, we perturbed this parameter and repeated the simulations leading to Figure 4 in the main text. The results are shown in Figure S2. As we can see, a two-fold reduction of θ (left figures) does not change the qualitative behavior of the model and the three energy scales (maintenance, switch to fermentation and maximum growth rate) are approximately the same as in Figure 4. on the other hand, a two-fold increase of θ (right figures) has only a quantitative effect on solutions where fermentation is active. However pure OxPhos becomes infeasible. In this case, the concomitant increase in ϕ_M is similar to increasing ϕ_0, since less cytoplasmic space is available for metabolic enzymes and the cell must switch to fermentation.

Some cell types have a high share of proteins without metabolic activity. In our model this is reflected in the parameter ϕ_0. The effect of varying this parameter on the growth rate is shown in Fig. 5 of the main text and Fig. S3 here. The dependence is qualitatively similar in both cases. In Fig. S3 we also show the maximum growth rate of the pure OxPhos solution and the solutions of maximum yield and minimum carbon uptake. These results are qualitatively similar up to $\phi_0 \approx 0.3$. Notably, pure OxPhos becomes infeasible at $\phi_0 = 0.4$. This implies that cells with an increased background share of non-metabolic proteins must switch to obligatory fermentation.

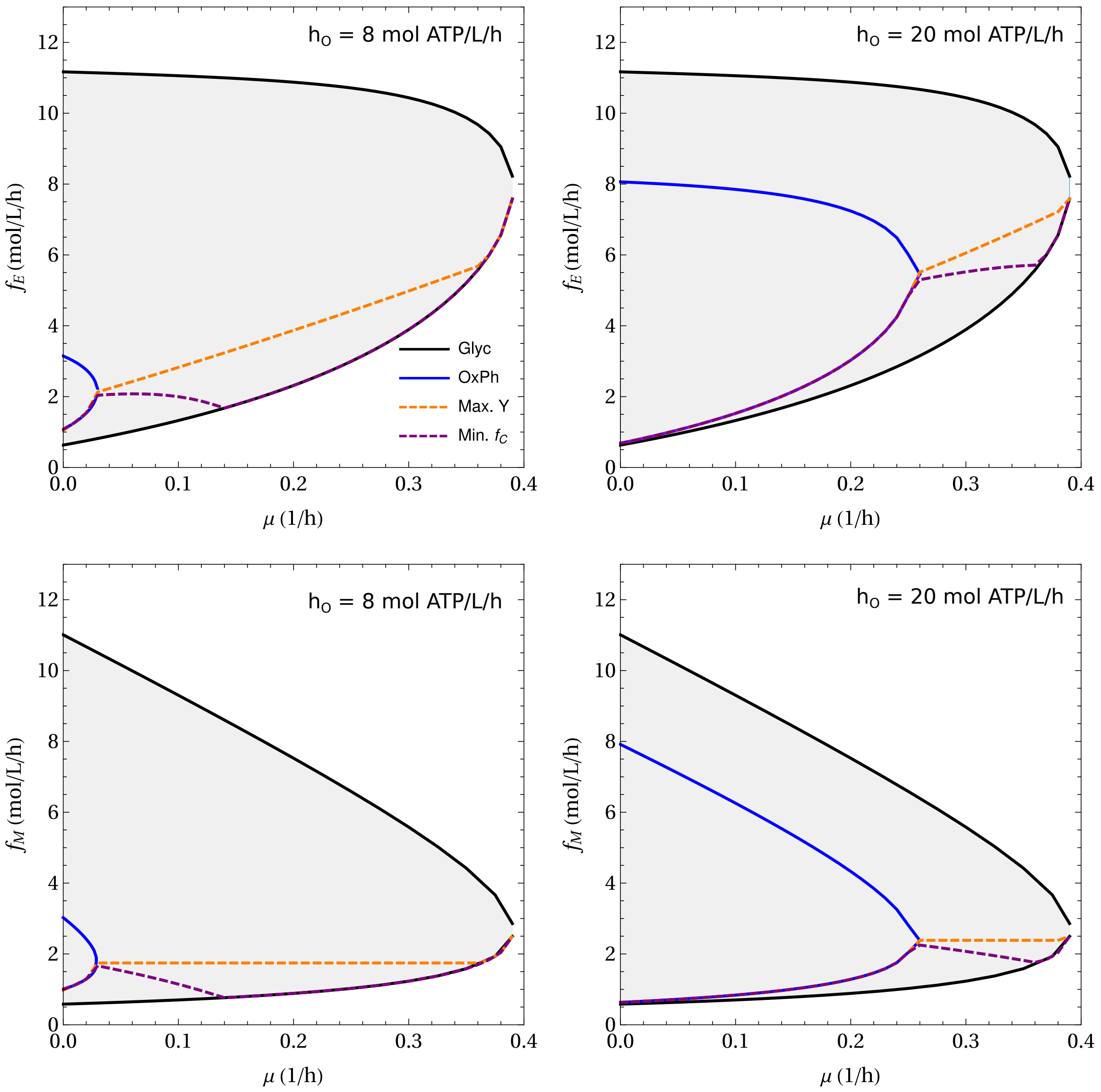

Figure S1: **Energy balance as a function of the growth rate for different values of h_O.** To assess the sensitivity of the model to variations in mitochondrial horsepower, we repeated the simulations of Figure 4 in the main text for different values of this parameter. See caption of Figure 4 for details.

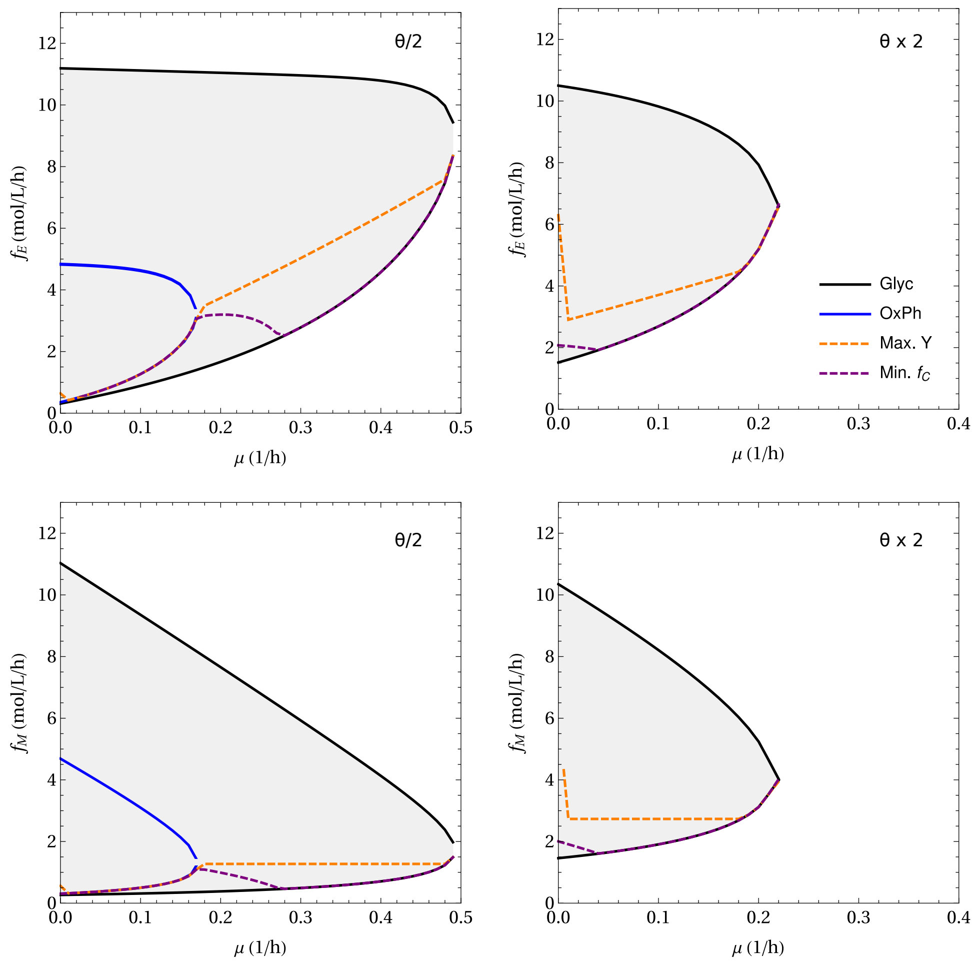

Figure S2: **Energy balance as a function of the growth rate for different values of $\theta = \frac{V_M}{V_c}\frac{1}{pFd}$.** To assess the sensitivity of the model to variations in the parameters of molecular motors, we repeated the simulations of Figure 4 in the main text for different values of θ. See caption of Figure 4 for details.

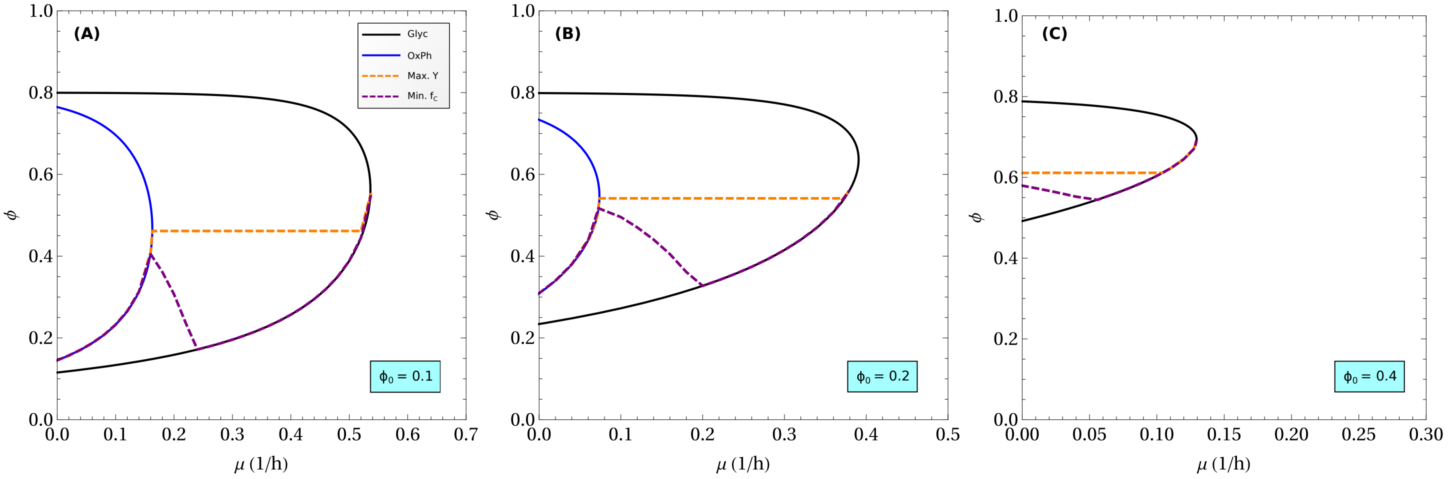

Figure S3: **Impact of the volume fraction of background proteins (extended).** Growth rate vs. ϕ for different values of ϕ_0. The blue curve is the maximum growth attainable with pure oxidative phosphorylation. The dashed range trajectory is the solution maximizing carbon yield. The dashed purple trajectory is the solution minimizing carbon use. At $\phi_0 = 0.4$ pure oxidative phosphorylation becomes infeasible.

Additional figures

Our model reproduces the linear dependence between ribosome density and growth rate found in other studies [4] (Figure S4).

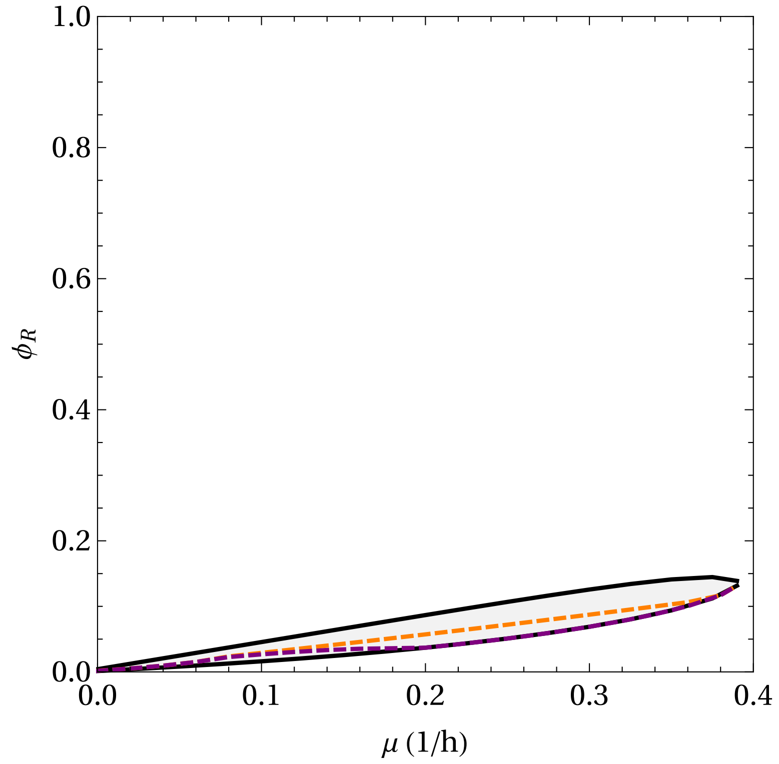

Figure S4: **Ribosomal volume fraction.** Feasible range (corresponding to varying ϕ) of the volume fraction occupied by ribosomes for different growth rates. The dashed lines show the trajectories corresponding to lowest carbon consumption (purple) and maximum growth yield (orange). These plots were obtained using the same parameters as Figure 4 in the main text.

The rate of fermentation at different temperatures was measured in Ref. [5] using an *in vitro* reconstitution of glycolytic enzymes. In Fig. S5 we fitted this data to the Arrhenius law $f = f_0 e^{-E_a/(RT)}$. The blue dot is the interpolated rate $f = 45\,\mu$mol/min/mL at $T = 37°$C used in the text to estimate h_F (see Methods).

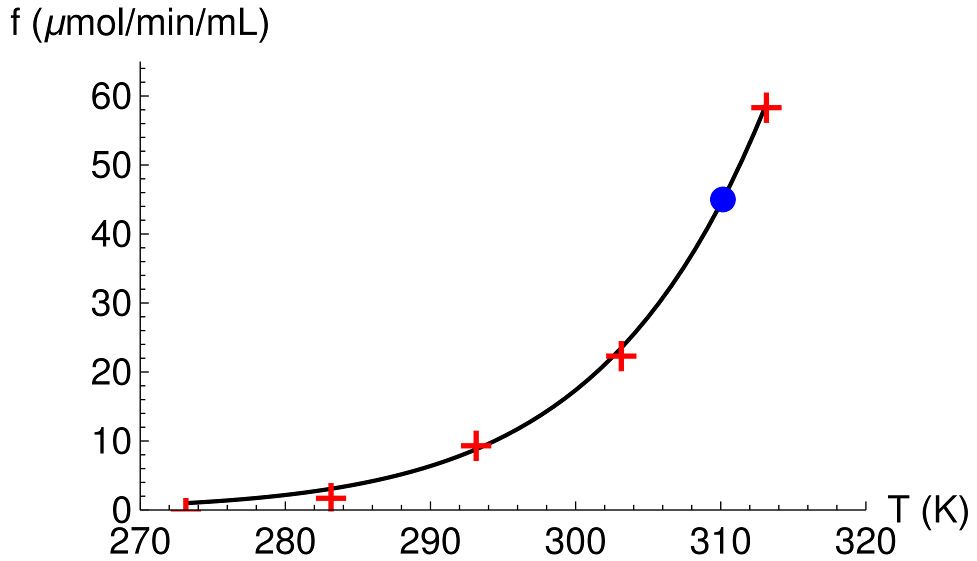

Figure S5: **Rate of glycolysis vs. temperature.** The red points are the rates of fermentation between 0 and $40°$C as measured in an *in vitro* reconstitution of glycolytic enzymes [5]. The curve is the Arrhenius law $f = f_0 e^{-E_a/(RT)}$, with fitted parameters $f_0 = 7.5 \times 10^{13}\,\mu$mol/min/mL, $E_a = 72.6\,$kJ/mol.

References

[1] Stephanie C. Weber, Andrew J. Spakowitz, and Julie A. Theriot. Nonthermal ATP-dependent fluctuations contribute to the in vivo motion of chromosomal loci. *Proceedings of the National Academy of Sciences*, 109(19):7338–7343, August 2012.

[2] BradleyR. Parry, IvanV. Surovtsev, MatthewT. Cabeen, CoreyS. OHern, EricR. Dufresne, and Christine Jacobs-Wagner. The bacterial cytoplasm has glass-like properties and is fluidized by metabolic activity. *Cell*, 156(1):183–194, 2014.

[3] Alexei Vazquez. *Overflow metabolism: from yeast to Marathon runners*. Academic Press, 2017.

[4] Markus Basan, Sheng Hui, Hiroyuki Okano, Zhongge Zhang, Yang Shen, James R. Williamson, and Terence Hwa. Overflow metabolism in escherichia coli results from efficient proteome allocation. *Nature*, 528(7580):99–104, 2015.

[5] Robert K. Scopes. Studies with a reconstituted muscle glycolytic system. the rate and extent of creatine phosphorylation by anaerobic glycolysis. *Biochemical Journal*, 134(1):197–208, 1973. 00070.

Table S1. Parameters used in the model. BNID identifiers refer to the BioNumbers database[1] (Milo et al. 2010).

Symbol	Name	Value	Units	Ref./Equation
v_s	specific excluded volume of macromolecules	2	mL/g	(Zimmerman and Trach 1991)
m_a	average amino acid mass	109	g/mol amino acid (aa)	BNID 104877, (Spahr 1962)
c_F, c_M, c_0	protein concentrations	4.6	mol aa/L	$1/(m_a v_s)$
c_R		4.8	mol aa/L	(Khatter et al. 2015)
v_{mito}	specific mitochondria volume per protein mass	2.6	mL/g	(Schwerzmann et al. 1989)
c_O	mitochondrial protein concentration	3.5	mol aa/L	$1/(m_a v_{mito})$
f_0	Arrhenius parameters of fermentation	7.5×10^{13}	μmol/min/mL	Fit to data of (Scopes 1973). See Figure S3.
E_F		72.6	kJ/mol	
E_R	Ribosome activation energy	80	kJ/mol	(Fehling and Weidner 1986)
E_M	Myosin activation energy	125	kJ/mol	(Bottinelli et al. 1996)
E_O	Acitvation energy of oxidative phosphorylation	50	kJ/mol	Estimated from activation energy of ATP synthase in yeast (Cobon and Haslam 1973)
h_F	fermentation horsepower	34	mol ATP/L/h	$f_0 e^{-E_F/RT}$, $T = 37$ °C
h_O	Oxidative phosphorylation horsepower	10	mol ATP/L/h	(Vazquez 2017)
h_R	Ribosome horsepower	8.4	mol aa/L/h	(Vazquez 2017)
F	Molecular motor kicking force	5	pN	(Clemen et al. 2005; Finer, Simmons, and Spudich 1994)
d	Molecular motor kicking step size	10	nm	(Clemen et al. 2005; Finer, Simmons, and Spudich 1994)
κ	Molecular motor kicking rate	5	1/s	(Pierobon et al. 2009; Milo and Phillips 2015)
τ	Molecular motor kick duration	0.5	s	(Pierobon et al. 2009; Milo and Phillips 2015)
M_M	Molecular motor molar mass	215405	g/mol	UNIPROT:Q9Y4I1
m_M	Maintenance energy per motor volume	42	mol/h/L	$\kappa/(M_M v_s)$

[1] http://bionumbers.hms.harvard.edu/

References cited in Table S1

Bottinelli, R., M. Canepari, M. A. Pellegrino, and C. Reggiani. 1996. “Force-Velocity Properties of Human Skeletal Muscle Fibres: Myosin Heavy Chain Isoform and Temperature Dependence.” *The Journal of Physiology* 495 (Pt 2) (September): 573–86.

Clemen, Anabel E-M, Mojca Vilfan, Johann Jaud, Junshan Zhang, Michael Bärmann, and Matthias Rief. 2005. “Force-Dependent Stepping Kinetics of Myosin-V.” *Biophysical Journal* 88 (6): 4402–10.

Cobon, G. S., and J. M. Haslam. 1973. “The Effect of Altered Membrane Sterol Composition on the Temperature Dependence of Yeast Mitochondrial ATPase.” *Biochemical and Biophysical Research Communications* 52 (1): 320–26.

Fehling, E., and M. Weidner. 1986. “Temperature Characteristics and Adaptive Potential of Wheat Ribosomes.” *Plant Physiology* 80 (1): 181–86.

Finer, J. T., R. M. Simmons, and J. A. Spudich. 1994. “Single Myosin Molecule Mechanics: Piconewton Forces and Nanometre Steps.” *Nature* 368 (6467): 113–19.

Khatter, Heena, Alexander G. Myasnikov, S. Kundhavai Natchiar, and Bruno P. Klaholz. 2015. “Structure of the Human 80S Ribosome.” *Nature* 520 (7549): 640–45.

Milo, Ron, Paul Jorgensen, Uri Moran, Griffin Weber, and Michael Springer. 2010. “BioNumbers--the Database of Key Numbers in Molecular and Cell Biology.” *Nucleic Acids Research* 38 (Database issue): D750–53.

Milo, Ron, and Rob Phillips. 2015. *Cell Biology by the Numbers*. Garland Science.

Pierobon, Paolo, Sarra Achouri, Sébastien Courty, Alexander R. Dunn, James A. Spudich, Maxime Dahan, and Giovanni Cappello. 2009. “Velocity, Processivity, and Individual Steps of Single Myosin V Molecules in Live Cells.” *Biophysical Journal* 96 (10): 4268–75.

Schwerzmann, K., H. Hoppeler, S. R. Kayar, and E. R. Weibel. 1989. “Oxidative Capacity of Muscle and Mitochondria: Correlation of Physiological, Biochemical, and Morphometric Characteristics.” *Proceedings of the National Academy of Sciences of the United States of America* 86 (5): 1583–87.

Scopes, R. K. 1973. “Studies with a Reconstituted Muscle Glycolytic System. The Rate and Extent of Creatine Phosphorylation by Anaerobic Glycolysis.” *Biochemical Journal* 134 (1): 197–208.

Spahr, P. F. 1962. “Amino Acid Composition of Ribosomes from Escherichia Coli.” *Journal of Molecular Biology* 4 (May): 395–406.

Vazquez, Alexei. 2017. *Overflow Metabolism: From Yeast to Marathon Runners*. Academic Press.

Zimmerman, S. B., and S. O. Trach. 1991. “Estimation of Macromolecule Concentrations and Excluded Volume Effects for the Cytoplasm of Escherichia Coli.” *Journal of Molecular Biology* 222 (3): 599–620.
